# Deep learning‐based motion correction algorithm for coronary CT angiography: Lowering the phase requirement for morphological and functional evaluation

**DOI:** 10.1002/acm2.14104

**Published:** 2023-07-24

**Authors:** Xiaoling Yao, Sihua Zhong, Maolan Xu, Guozhi Zhang, Yuan Yuan, Tao Shuai, Zhenlin Li

**Affiliations:** ^1^ Department of Radiology West China Hospital of Sichuan University Chengdu China; ^2^ United Imaging Healthcare Shanghai China

**Keywords:** coronary artery disease, coronary computed tomography angiography, deep learning, motion correction

## Abstract

**Purpose:**

To investigate the performance of a deep learning‐based motion correction algorithm (MCA) at various cardiac phases of coronary computed tomography angiography (CCTA), and determine the extent to which it may allow for reliable morphological and functional evaluation.

**Materials and methods:**

The acquired image data of 53 CCTA cases, where the patient heart rate (HR) was ≥75 bpm, were reconstructed at 0, ±2, ±4, ±6, and ±8% deviations from each optimal systolic phase, with and without the MCA, yielding a total of 954 images (53 cases × 9 phases × 2 reconstructions). The overall image quality and diagnostic confidence were graded by two radiologists using a 5‐point scale, with scores ≥3 being deemed clinically interpretable. Signal‐to‐noise ratio, contrast‐to‐noise ratio, vessel sharpness, and circularity were measured. The CCTA‐derived fractional flow reserve (CT‐FFR) was calculated in 38 vessels on 24 patients to identify functionally significant stenosis, using the invasive fractional flow reserve (FFR) as reference. All metrics were compared between two reconstructions at various phases.

**Results:**

Inferior image quality was observed as the phase deviation was enlarged. However, MCA significantly improved the image quality at nonoptimal phases and the optimal phase. Coronary artery evaluation was feasible within 4% phase deviation using MCA, with interpretable overall image quality and high diagnostic confidence. With MCA, the performance of identifying functionally significant stenosis via CT‐FFR was increased for images at various phase deviations. However, obvious decrease in accuracy, as compared to the image at the optimal phase, was found on those with deviations >4%.

**Conclusion:**

The deep learning‐based MCA allows up to 4% phase deviation in acquiring CCTA for reliable morphological and functional evaluation on patients with high HRs.

## 1 INTRODUCTION

Coronary artery disease (CAD) is a leading cause of mortality and morbidity in the majority of the world's population.^1^ Current guidelines^2^ recommend coronary computed tomography angiography (CCTA) as a front‐line diagnostic strategy for CAD assessment. However, the presence of motion artifacts, which is commonly found on CCTA, may induce image quality degradation, challenging the accuracy of CCTA‐based clinical interpretation especially for patients with high heart rate (HR). In this regard, a scanner with wider z‐coverage and faster rotation is helpful for motion artifact reduction, since it offers a better temporal resolution.^3^ Algorithmic motion correction is another solution that is supposed to be more accessible, for its software nature.^4^, ^5^ Such motion correction algorithms (MCAs) generally consist of one motion estimation and one compensation step. More recently, deep learning techniques were also introduced to the development of MCAs, where favorable motion correction can be obtained with less computing power once the model has been trained.^6^

The efficiency of MCAs in reducing motion artifacts in patients with insufficient HR control has been extensively demonstrated by previous studies.^7^, ^8^, ^9^ However, these results were limited by the fact that only the optimal cardiac phase with the least motion was evaluated. Regardless of the nature of method, for example, empirical, semi‐automatic, or automatic, identifying the optimal cardiac phase is a process of estimation due to the complexity of cardiac motion.^10^ It is not rare in clinical reality that the selected phase for CCTA evaluation does not fully coincide with the absolute optimal phase in theory. With MCAs becoming one of the routine measures against high HR, it is also of interest to know whether they can make the images at nonoptimal cardiac phases equally useful, or in other words, lower the requirement on determining the optimal phase. In this study, we hypothesized that the MCAs are able to deal with the motion artifacts on CCTA images within a certain degree of phase deviation, providing sufficient image quality as commonly achievable at the optimal cardiac phase.

Accordingly, to test this hypothesis, a commercially available deep learning‐based MCA that is routinely used in our institution was applied. By investigating the performance of the MCA at various cardiac phases, the study aims to determine the extent to which the MCA may allow for reliable morphological and functional evaluation of CAD in patients with HR ≥ 75 bpm. To this end, multiphase systolic CCTA images were extracted from patients with high HR (HR ≥ 75 bpm). A side‐by‐side comparison was made between images with and without the MCA, in both quantitative and qualitative manner for morphological assessment. The impact on functional evaluation was characterized by comparing the performance of identifying functionally significant CAD via CCTA‐derived fractional flow reserve (CT‐FFR) with respect to the invasive fractional flow reserve (FFR) as reference.

## 2 MATERIALS AND METHODS

### 2.1 Patient population

This retrospective study was approved by the Medical Research Ethical Committee of our institution. Between February and July 2022, 175 consecutive patients with suspected or known CAD who underwent CCTA were screened for inclusion in this study. Among them, 122 patients were excluded: (1) HR < 75 bpm (48 patients); (2) history of coronary revascularization (12 patients); (3) recently presented with acute coronary syndrome (3 patients), and (4) lack of CT raw data (59 patients), which is needed for MCA reconstruction. Therefore, the final study population consisted of 53 patients.

### 2.2 Deep learning‐based motion correction algorithm

The MCA (CardioCapture^®^, United Imaging Healthcare) used in this study is a novel cardiac motion estimation and compensation algorithm, which incorporates a deep convolutional neural network (DCNN) into the image reconstruction workflow.^9^ The DCNN was trained with diverse coronary artery images in order to obtain a generalized learning such that the coronary arteries at cardiac phases adjacent to the targeted phase, that is, the phase to be corrected, can be precisely segmented in the projection domain. Using information from those coronary artery trees segmented by DCNN, the MCA estimates the coronary artery motion and generates a vessel motion vector for motion compensation at the targeted cardiac phase.

### 2.3 CCTA acquisition and image reconstruction

All CCTA examinations were carried out on a 320‐row CT scanner (uCT 960+, United Imaging Healthcare) with a prospective ECG‐triggered axial scan mode. Scanning parameters were as follows: Tube voltage was 100/120 kVp with auto‐kV switched on (≤25 kg/m^2^, 100 kVp; >25 kg/m^2^, 120 kVp), and tube current was determined by dose modulation technique; Gantry rotation time was 0.25 s; and collimation was 320×0.5 mm. The acquisition window was limited between 30 and 85% of the R‐R interval. Iodinated contrast medium (Iomeprol 400 mgI/mL, Bracco Imaging Italia S.r.L.) with a weight‐dependent volume of 0.8 kg/mL was administered at an injection rate of 5.0 mL/s. The injection duration was 10 s for all patients and the delivery of contrast medium was followed by 30‐mL saline solution at the same injection rate. Bolus tracking was set relative to the level of the proximal descending aorta, and data acquisition was triggered 2 s after a threshold of 220 HU was achieved. All included patients followed their baseline medications, where additional Beta‐blocker was not given before CCTA scanning.

The optimal systolic phase for each enrolled patient was determined by consensus among two experienced cardiovascular radiologists (Y.Y., T.S.). For the purpose of this study, multiphase images were reconstructed at 0, ±2, ±4, ±6, and ±8% deviations with respect to the optimal phase, using a standard reconstruction algorithm (SRA) and additionally with the MCA. All reconstructions were set with 0.5‐mm slice thickness and 0.5‐mm slice interval. Finally, a total of 954 image datasets (53 patients × 9 phases × 2 reconstructions) were available for analysis.

### 2.4 Image quality assessment

**Qualitative image quality**–All images were independently analyzed by the two aforementioned radiologists (Y.Y., T.S.), with fully blinded patient demographics and reconstruction methods. The images with and without MCA reconstruction were randomly presented with an interval of 2 weeks to minimize any potential reader bias. The radiologists performed qualitative analysis in terms of diagnostic confidence and overall image quality using an 18‐segment model for vessels ≥1.5 mm in diameter. Accordingly, the diagnostic confidence for identifying any significant stenosis ≥50% was evaluated using a 5‐point scale, where 1 = poor; 2 = low; 3 = average; 4 = high, and 5 = excellent. The overall image quality, including regions beyond the coronary arteries and factors such as noise appearance and beam‐hardening artifacts, was also assessed on a 5‐point scale, where 1 = poor, 2 = low, 3 = average, 4 = good, and 5 = excellent. If the discrepancy was raised, consensus was made by negotiation between the two radiologists. The image was only considered interpretable if scores on both diagnostic confidence and overall image quality were ≥3.

**Quantitative image quality**–Another cardiovascular radiologist (X.Y.) was asked to delineate regions of interest (ROIs) for quantitative measurement. ROIs were placed on the chest wall fat at the level of the aortic root, as well as on the proximal right coronary artery (RCA), left anterior descending artery (LAD), and left circumflex artery (LCX). Care was taken to avoid adjacent structures in each ROI. Image noise was defined as the standard deviation of attenuation measured in a ROI. Signal‐to‐noise ratio (SNR) and contrast‐to‐noise ratio (CNR) were calculated as follows:

$$SNR\;=\;\frac{\;{\bar{HU}}_{vessel}}{\;{\bar{SD}}_{vessel}}\text{and}\;CNR\;=\;\frac{{\bar{HU}}_{vessel}\;-HU_{fat}}{SD_{fat}}$$

where ${\bar{HU}}_{vessel}$ and ${\bar{SD}}_{vessel}$ refer to the mean attenuation and image noise, respectively, for ROIs placed on the proximal RCA, LAD, and LCX. HU_fat_ and SD_fat_ represent the attenuation and image noise, respectively, for ROIs placed on the chest wall fat.

**FIGURE 1:**
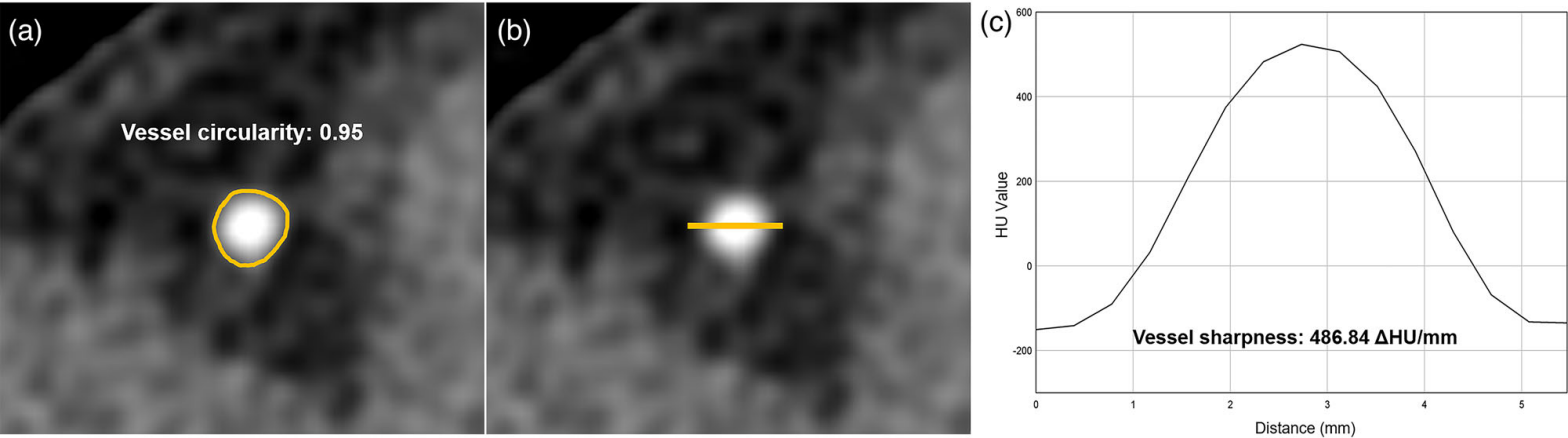
Examples of quantitative measurement in our study. (a) Vessel circularity measurement. (b) Vessel sharpness measurement. (c) Profile curve for vessel edge.

Furthermore, quantitative measurements of vessel sharpness and circularity, both of which reflect the degree of cardiac motion, were obtained (Figure 1). Vessel sharpness was evaluated on the proximal RCA, LAD, and LCX. For each image, a line was drawn perpendicular to the coronary arteries to generate line profiles of the vessels using an open‐source software (ImageJ, National Institutes of Health, Maryland, USA). All pixel intensities along the generated lines were recorded, and regression lines for vessel border were derived. Then, vessel sharpness was computed as the mean value of the maximum slopes of the regression lines for RCA, LAD, and LCX. Vessel circularity was quantified following a previously described method.^11^ Briefly, through‐plane vessel regions were manually segmented, and vessel circularity was calculated as $\frac{P^{2}}{4\pi A}$, where *P* and *A* denote the perimeter and area of the segmented vessel region, respectively. Vessel circularity measurements were computed for RCA, LAD, and LCX, and then averaged to yield a mean vessel circularity for each image.

### 2.5 CCTA‐derived fractional flow reserve assessment

CT‐FFR is a well‐established functional imaging technique for non‐invasive assessment of hemodynamically significant stenosis causing ischemia.^12^, ^13^ Among 53 patients included in this study, 24 were further referred for invasive coronary angiography (ICA) and invasive FFR measurement within 2 months after CCTA, both of which were performed in accordance with standard practice guidelines and regulations.^2^ Lesions with FFR values ≤0.8 were considered hemodynamically significant stenosis.

CT‐FFR calculations were performed for these 24 patients using a validated software tool (uCT‐FFR version 1.5, United Imaging Healthcare)^14^ via the following steps: (1) three‐dimensional reconstruction of the coronary arteries; (2) vessel centerline definition; (3) boundary condition estimation, and (4) CT‐FFR calculation. Vessel centerline and luminal contours for the entire coronary artery tree were automatically generated, on which further manual adjustment was performed as needed.

CT‐FFR values obtained from the distal to the target stenosis were recorded by the fourth radiologist (M.X.) who was blinded to the patient demographics. The location to measure CT‐FFR was consistent across different phase images for each patient. Functionally significant stenosis was defined as CT‐FFR value ≤0.8. In addition, cases with score 1 in an overall image quality were unable to reconstruct complete or reliable model of the coronary artery for CT‐FFR calculation and was conservatively classified ischemic, following an intent‐to‐diagnose manner. Using the invasive FFR as reference, the diagnostic performance in terms of sensitivity, specificity, accuracy, and area under the receiver–operator characteristic curve (AUC) were calculated to assess the discrimination of functionally significant stenosis by CT‐FFR.

### 2.6 Statistical analysis

All statistical analyses were conducted using SPSS Version 20.0 (IBM Corp., Armonk, USA) and MedCalc Version 19.8 (MedCalc Software Ltd., Ostend, Belgium). Continuous variables were presented as mean ± standard deviation, while categorical variables were described as numbers and percentages. The normality of quantitative data was examined by the Kolmogorov–Smirnov test. Quantitative and qualitative data were compared between any given pair of studied groups by the Wilcoxon signed‐rank test or the paired *t* test, wherever it applies. The chi‐square test was used to test the difference in the interpretability between images with and without MCA. Interobserver agreement for qualitative evaluation was tested by the Cohen's Kappa analysis. AUCs were compared by the DeLong test. A *p* value < 0.05 was denoted statistically significant.

## 3 RESULTS

### 3.1 Patient characteristics

**FIGURE 2:**
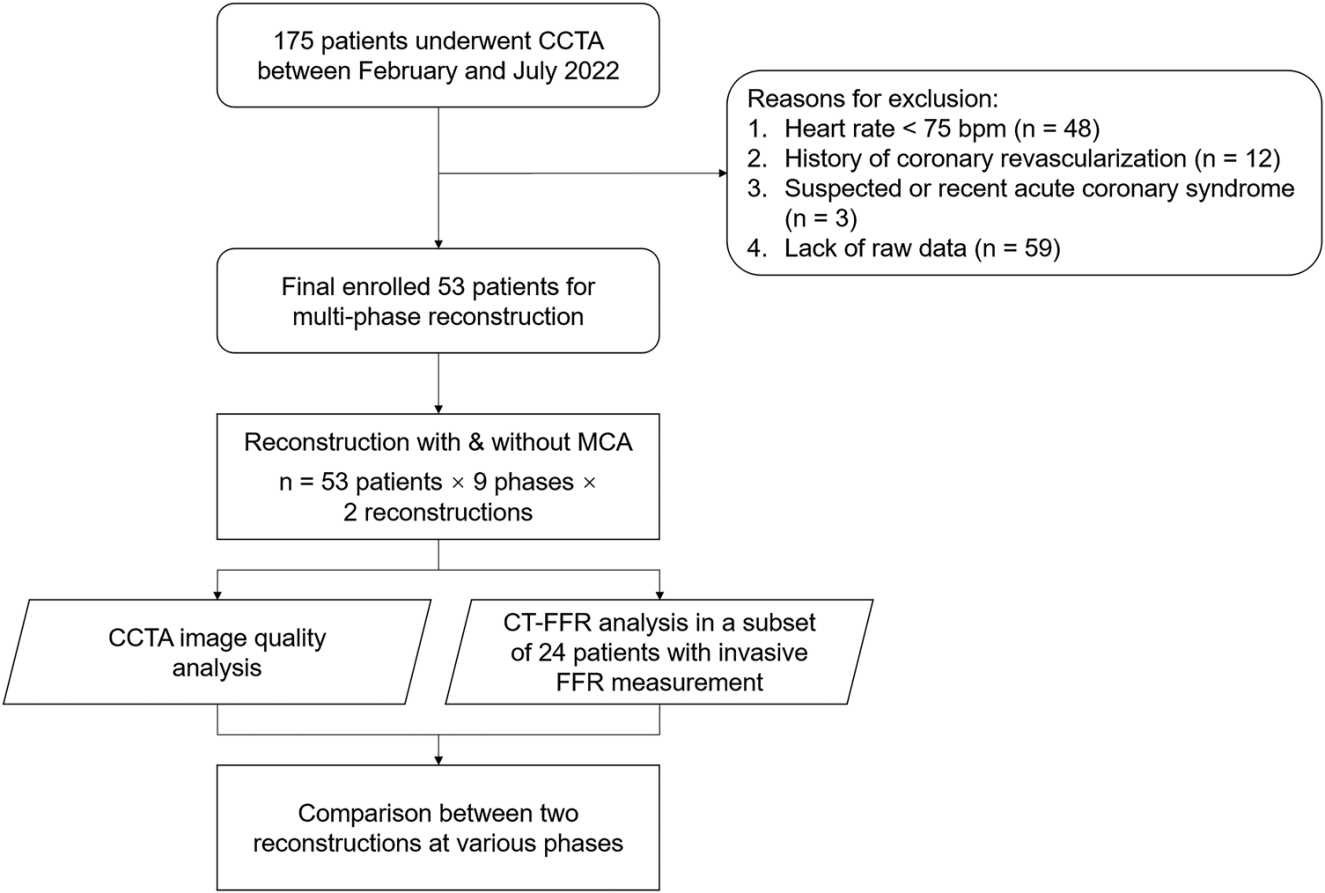
Flowchart of the study design.

Among the 53 patients (26.4% female) enrolled in this study, the mean age was 75.61 ± 8.74 years, the mean body mass index was 23.29 ± 3.21 kg/m^2^, the mean HR during scanning was 89.10 ± 12.52 bpm, and the mean HR variability was 11.30 ± 18.70 bpm. With 2% interval reconstruction, the mean optimal systolic phase was found at 47.61 ± 6.29%. Detailed information of patient characteristics is shown in Table 1. The study design is illustrated in Figure 2.

**TABLE 1:** Patient characteristics.

Characteristics (*n* = 53)	
Age (years)	75.61 ± 8.74
Female (%)	14 (26.4%)
Body mass index (kg/m^2^)	23.29 ± 3.21
Heart rate (bpm)	89.10 ± 12.52
Heart rate variability (bpm)	11.30 ± 18.70
Risk factors	
Diabetes mellitus (%)	17 (32.1%)
Hypertension (%)	28 (52.8%)
Smoking (%)	10 (18.9%)
Family history of CAD (%)	6 (11.3%)
Agatston score	289.31 ± 353.19

Abbreviation: CAD, coronary artery disease.

### 3.2 Qualitative image quality analysis

Good interobserver agreement for image quality scores was achieved, with Kappa ranging from 0.63 to 0.71. In our study, the qualitative image quality for images reconstructed with and without MCA were significantly deteriorated by the enlargement of phase deviation. Nevertheless, the qualitative metrics in terms of diagnostic confidence and overall image quality were significantly improved by applying MCA (Table 2). Specifically, the improvement was more pronounced at 4% phase deviation, with the mean difference for diagnostic confidence and overall image quality of 0.38 and 0.62, respectively. After the use of MCA within 8% phase deviation, the percentage of interpretable phases significantly increased from 37.53% (179/477) to 58.28% (278/477).

**TABLE 2:** Results of qualitative image quality scores.

	Diagnostic confidence			Overall image quality		
	SRA	MCA	*p*	SRA	MCA	*p*
Optimal phase	4.52 ± 0.83	4.76 ± 0.62	0.002	3.83 ± 0.75	4.21 ± 0.65	<0.001
2% phase deviation	4.17 ± 0.91	4.55 ± 0.62	<0.001	3.1 ± 0.75	3.58 ± 0.61	<0.001
4% phase deviation	3.76 ± 0.93	4.14 ± 0.9	<0.001	2.46 ± 0.85	3.08 ± 0.71	<0.001
6% phase deviation	3.26 ± 0.91	3.6 ± 0.96	<0.001	1.77 ± 0.72	2.29 ± 0.85	<0.001
8% phase deviation	2.83 ± 0.93	3.19 ± 0.99	<0.001	1.4 ± 0.57	1.71 ± 0.85	<0.001

Abbreviations: MCA, motion correction algorithm; SRA, standard reconstruction algorithm.

### 3.3 Quantitative image quality analysis

**FIGURE 3:**
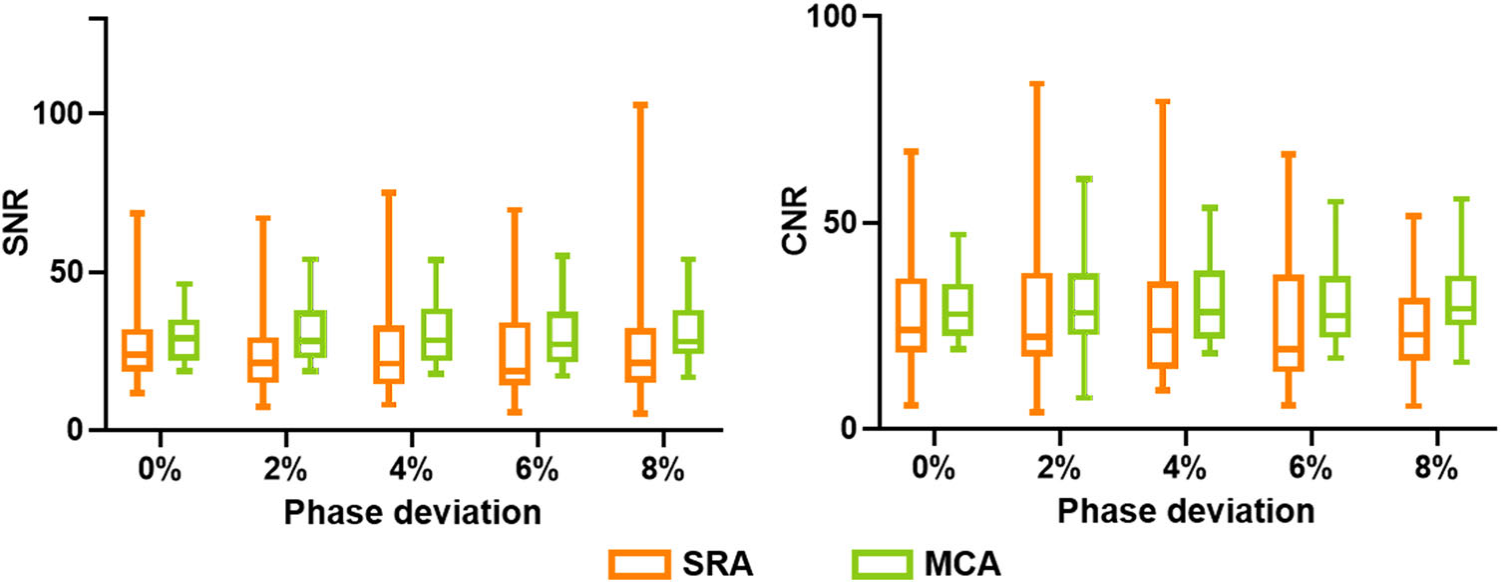
Results of SNR and CNR analysis. CNR, contrast‐to‐noise ratio; SNR, signal‐to‐noise ratio.

The RCA, LAD, and LCX in all images exhibited a mean attenuation of exceeding 300 HU, which was considered adequate enhancement for vessel visualization. Results of SNR and CNR can be seen in Figure 3. Neither the reconstruction methods nor the selected phases had a significant influence on SNR and CNR.

Vessels associated with higher degree of motion presented a more blurring, less circular edge. According to our quantitative analysis, the vessel sharpness and circularity significantly decreased as the phase deviation was enlarged, which was reasonable since the optimal phase was expected to have the least motion. Despite that, motion artifacts were substantially reduced by MCA within 8% phase deviation. Moreover, MCA was found particularly effective in suppressing moderate‐to‐severe motion artifacts, as revealed by the superior improvement on vessel sharpness and circularity at 4 and 6% phase deviation (Table 3).

**TABLE 3:** Results of vessel sharpness and circularity analysis.

	Vessel sharpness (ΔHU/mm)			Vessel circularity		
	SRA	MCA	*p*	SRA	MCA	*p*
Optimal phase	382.61 ± 121.45	400.1 ± 117.68	0.003	0.89 ± 0.06	0.9 ± 0.05	0.001
2% phase deviation	373.06 ± 129.44	381.54 ± 123.11	0.626	0.87 ± 0.08	0.89 ± 0.07	0.04
4% phase deviation	350.06 ± 119.05	373.53 ± 127.49	0.001	0.84 ± 0.1	0.87 ± 0.08	0.001
6% phase deviation	297.37 ± 108.4	334.46 ± 127.15	0.001	0.8 ± 0.12	0.85 ± 0.1	<0.001
8% phase deviation	291.12 ± 114.38	313.87 ± 120.92	0.01	0.77 ± 0.13	0.79 ± 0.13	0.009

Abbreviations: MCA, motion correction algorithm; SRA, standard reconstruction algorithm.

### 3.4 Diagnostic performance of CT‐FFR

A total of 38 vessels measured by both ICA and invasive FFR from 24 patients were considered for the CT‐FFR evaluation. As indicated by the ICA, 7 vessels were found with stenosis in degree of 25%−49%, 21 vessels with stenosis of 50%−69%, and 10 vessels with stenosis of 70%−99%. As indicated by invasive FFR, a total of 16 vessels (42.11%) were identified ischemic.

**FIGURE 4:**
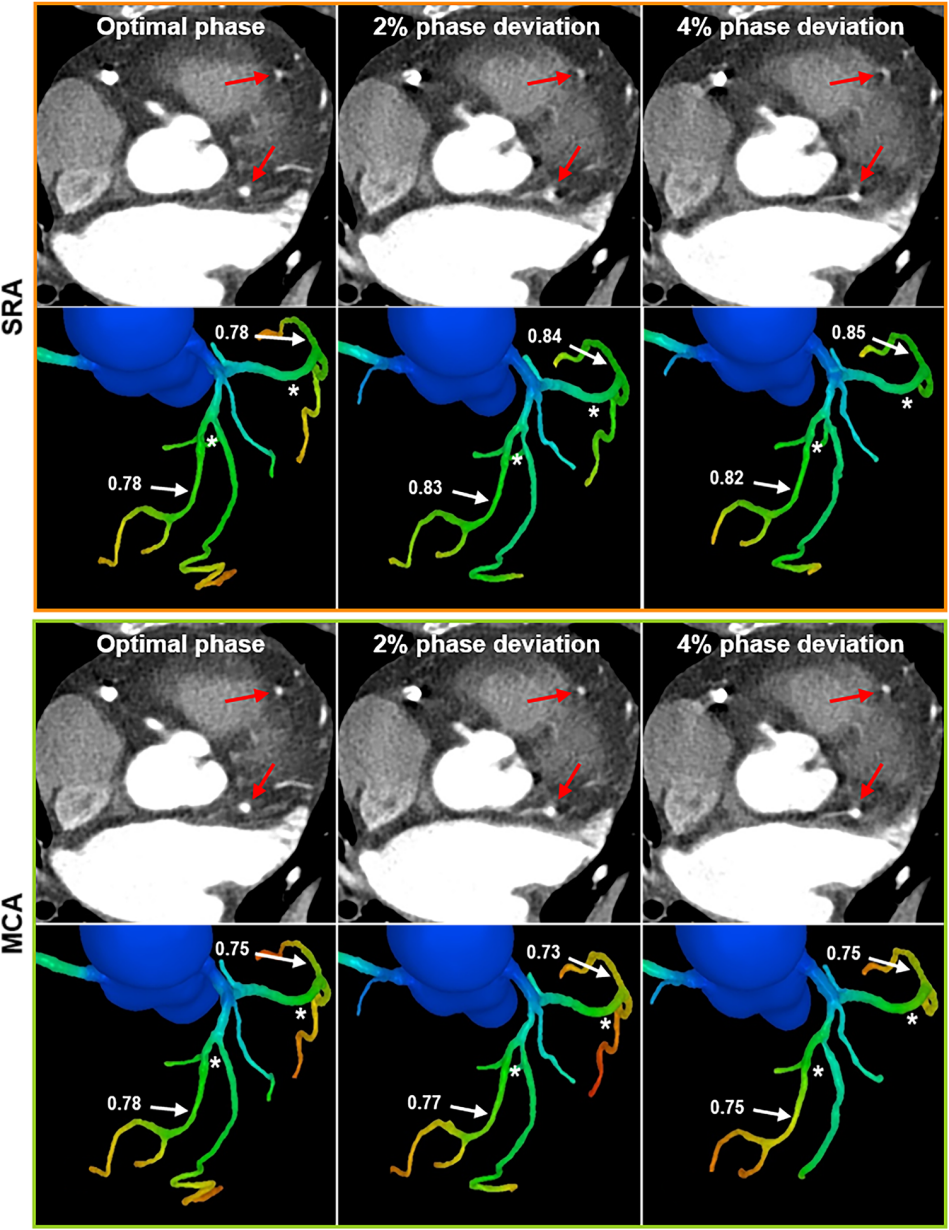
A 75‐year‐old female patient with typical chest pain referred to ICA and invasive FFR measurement following CCTA. ICA confirmed moderate stenosis in the proximal LAD and LCX (white asterisk) with a FFR value of 0.78 and 0.77, respectively. CT‐FFR analysis at the optimal phase (white arrowhead) also revealed that the lesions were functionally significant stenosis. Without MCA, CT‐FFR changed from being significant to nonsignificant (>0.8) at 2 and 4% phase deviation. By applying the MCA, motion artifacts were substantially suppressed (red arrowhead) in the CCTA images and the CT‐FFR at up to 4% phase deviation were kept well consistent with invasive FFR. CCTA, coronary computed tomography angiography; CT‐FFR, CCTA‐derived fractional flow reserve; FFR, fractional flow reserve; ICA, invasive coronary angiography; LAD, left anterior descending artery; LCX, left circumflex artery; MCA, motion correction algorithm.

Table 4 shows the diagnostic performance for identifying functionally significant stenosis on SRA and MCA. There was no significant difference for the mean CT‐FFR value and AUC between SRA and MCA images at the optimal phase (CT‐FFR: 0.77 ± 0.12 vs. 0.79 ± 0.1; AUC: 0.83 vs. 0.86; All *p* > 0.05). Compared to SRA images, MCA exhibited better AUC at nonoptimal phases (All *p* < 0.05). A trend of reduced diagnostic performance was observed when the phase deviation was enlarged, while they were not statistically different within 4% phase deviation for MCA images. A representative case can be seen in Figure 4.

**TABLE 4:** Diagnostic performance of CT‐FFR with MCA versus SRA.

	Sensitivity (%)		Specificity (%)		Accuracy (%)		AUC	
	SRA	MCA	SRA	MCA	SRA	MCA	SRA	MCA
Optimal phase	94 (79–99)	94 (79–99)	73 (57–85)	77 (62–89)	82 (71–90)	84 (74–92)	0.83 (0.73–0.91)	0.86 (0.76–0.93)
2% phase deviation	91 (75–98)	94 (79–99)	57 (41–72)	77 (62–89)	71 (60–81)	84 (74–92)	0.74 (0.62–0.83)	0.86 (0.76–0.93)
4% phase deviation	84 (67–95)	91 (75–98)	48 (32–63)	77 (62–89)	63 (51–74)	83 (73–91)	0.66 (0.54–0.77)	0.84 (0.74–0.91)
6% phase deviation	94 (79–99)	97 (84–99)	36 (22–52)	55 (39–70)	61 (49–72)	72 (61–82)	0.65 (0.53–0.76)	0.75 (0.65–0.85)
8% phase deviation	94 (79–99)	100 (89–99)	36 (22–52)	43 (28–59)	61 (49–72)	67 (55–77)	0.65 (0.53–0.76)	0.72 (0.6–0.81)

*Note*: Values in parentheses are 95% confidence interval.

Abbreviations: AUC, area under the curve; MCA, motion correction algorithm; SRA, standard reconstruction algorithm.

With SRA, 60 cases from 16 vessels could be misclassified by CT‐FFR due to motion artifacts. Forty‐four out of 60 cases were correctly reclassified after additional reconstruction with the MCA. Among the 16 vessels, three vessels had the mean invasive FFR value of 0.77 ± 0.02, falling in the gray zone of FFR classification. Eight cases were misclassified as nonischemic with SRA, while only two cases were misclassified with MCA.

## 4 DISCUSSION

To our knowledge, this is the first study to look into the motion correction on CCTA at various cardiac phases from the perspective of both morphological and functional evaluation. In this study, we found that the deep learning‐based MCA was effective in reducing motion artifacts within 8% phase deviation from the optimal phase. Although inferior qualitative scores were reported as the phase deviation was enlarged, the MCA allows for the evaluation of coronary arteries with interpretable overall image quality and high diagnostic confidence within 4% phase deviation. Importantly, by applying the MCA, the diagnostic performance for identifying hemodynamic significance CAD at 2 and 4% phase deviations can be improved to a level similar to that at the optimal phase.

Cardiac motion artifact has been the primary challenge for CCTA imaging, with up to 12% of coronary segments falling below the common diagnostic acceptance level.^15^, ^16^ With various MCAs becoming routinely available, such as the Snapshot Freeze (SSF, GE Healthcare), the Adaptive Motion Correction (AMC, Cannon Medical System), and the CardioCapture as used in this study, a large number of studies have reported their efficiency for reducing residual motion artifacts at the optimal phase.^7^, ^8^, ^9^, ^17^ In this regard, the present study is consistent with these findings, showing that the MCA significantly improves both quantitative and qualitative image quality at the optimal phase. Another related study by Suh et al.^18^ has further proven that SSF extends motion correction to the whole heart in a single cardiac cycle, improving the visualization of aortic valve abnormalities. However, these investigations focused mainly on the impact of motion correction on morphological evaluation, while functional evaluation has not been explored, which is a key difference between our study and the others. It has been previously demonstrated that MCAs were able to improve the diagnostic accuracy of obstructive stenosis detection as compared with ICA.^8^, ^19^, ^20^, ^21^ As a further benefit, our study showed that the use of MCA appears to improve the reliability of CT‐FFR, particularly given the overwhelming evidence suggesting its clinical value in detecting hemodynamically significant stenosis.^22^, ^23^

With increasing HR, the length of diastole progressively decreases, while that of systole is less dependent on HR. Several studies have been conducted to analyze the HR dependency of motion artifacts, and also reported that preferred CCTA images for patients with high HR can be reconstructed in systole rather than diastole.^24^, ^25^ Therefore, cardiac phases in systole were selected in this study. Furthermore, we identified images within 8% phase deviation from the optimal phase for analysis to obtain a generalized conclusion. Based on the CCTA assessment results, we showed that the MCA used in this study has a significant impact on reducing motion artifacts at the optimal phase, as well as nonoptimal phases within 8% phase deviation. Notably, the mean qualitative scores were ≥3 within 4% phase deviation for MCA images, which are adequate for coronary artery evaluation, as reported in published literature.^26^, ^27^, ^28^

CT‐FFR is one of the noninvasive functional imaging that has been recommended by the current guidelines for ischemic CAD discrimination.^2^ CT‐FFR accuracy largely depends on the quality of input CCTA image, particularly for the depiction of vascular structures that may have also been associated with motion artifacts. While Leipsic et al.^29^ have shown that motion artifacts did not influence the performance of CT‐FFR, they did not systemically assess the degree of motion artifacts. In contrast, our study showed no significant differences on the diagnostic performance of CT‐FFR calculated from MCA images within 4% phase deviation, despite an inferior overall image quality being found at nonoptimal phases compared with the optimal phase. This discrepancy may be attributed to the principle of CT‐FFR model. CT‐FFR is determined by solving numerous complex equations that simulate the flow distribution of the model considering not only coronary artery segments but also patient‐specific information.^30^ Given the complexity of CT‐FFR modeling, CT‐FFR calculation is less affected by the segmentation error of coronary lumen caused by minor motion artifacts. The present findings suggested that the use of MCA reduces motion artifacts and the degradation of vascular depiction, offering sufficient CCTA image quality for obtaining accurate CT‐FFR within 4% phase deviation. In other words, MCA is necessary when calculating CT‐FFR from images at nonoptimal phases or in cases where the determination of the optimal phase might be uncertain.

Our findings are of clinical relevance, as the use of the MCA improves the feasibility and reliability for morphological and functional evaluation at 4% phase deviation to a similar level as for those at the optimal phase. This may compensate for diagnostic difficulties resulting from the inaccurate identification of the optimal phase, thereby promising the diagnostic performance of CAD, especially for patients with high HR.

It should be noted that the performance of data‐driven algorithms such as MCA could be easily affected by the characteristics of training samples.^31^ While reviewing the reconstructed images, readers reported that vascular distortions were observed in one case reconstructed with MCA, which caused an increase in the severity of luminal stenosis, although CT‐FFR values were similar between images with and without MCA reconstruction (Figure S1 in ESM). Further investigations are warranted to evaluate its performance at different clinical scenarios.

Our study had several limitations. First, this investigation was limited to one vendor‐specific MCA, where the results may or may not be translated to other algorithms and scanners. However, the demonstrated investigative strategy for determining the performance of motion correction should be directly applicable to other settings, which is more essential than the difference between MCAs. Second, a 2% phase interval was selected in characterizing the deviation, which could certainly also be set in smaller steps for a more detailed investigation on phase. Moreover, the optimal cardiac phase was subjectively determined by the readers, which may potentially introduce bias to the conclusions. Finally, we did not evaluate the impact of motion artifacts on the interpretation of plaques. It is within future plan to assess whether the MCA could improve the assessment of coronary calcium scores, plaque volume, and plaque types (i.e., calcified, noncalcified, or mixed), which could help predicting the risk of adverse cardiac events.^32^, ^33^

## 5 CONCLUSION

The deep learning‐based MCA as investigated in this study allows up to 4% phase deviation in acquiring CCTA for reliable morphological and functional evaluation on patients with high HR.

## AUTHOR CONTRIBUTIONS

*Conceptualization and study design*: Zhenlin Li and Guozhi Zhang. *Data collection*: Xiaoling Yao, Maolan Xu, and Tao Shuai. *Statistical analysis and data interpretation*: Xiaoling Yao, Sihua Zhong, Maolan Xu, Yuan Yuan, and Tao Shuai. *Manuscript preparation*: Xiaoling Yao, Sihua Zhong, and Guozhi Zhang; All authors read and approved the final manuscript.

## CONFLICT OF INTEREST SATEMENT

The authors declare no conflicts of interest.

## Supporting information

Supporting Information
