# Supplementary material for: Deep learning‐based motion correction algorithm for coronary CT angiography: Lowering the phase requirement for morphological and functional evaluation
Source: J Appl Clin Med Phys. 2023 Jul 24;24(9):e14104. doi: 10.1002/acm2.14104 (PMC10476979; doi:10.1002/acm2.14104)
Supplement: Supplementary file 1 — Supporting Information [file ACM2-24-e14104-s001.docx]

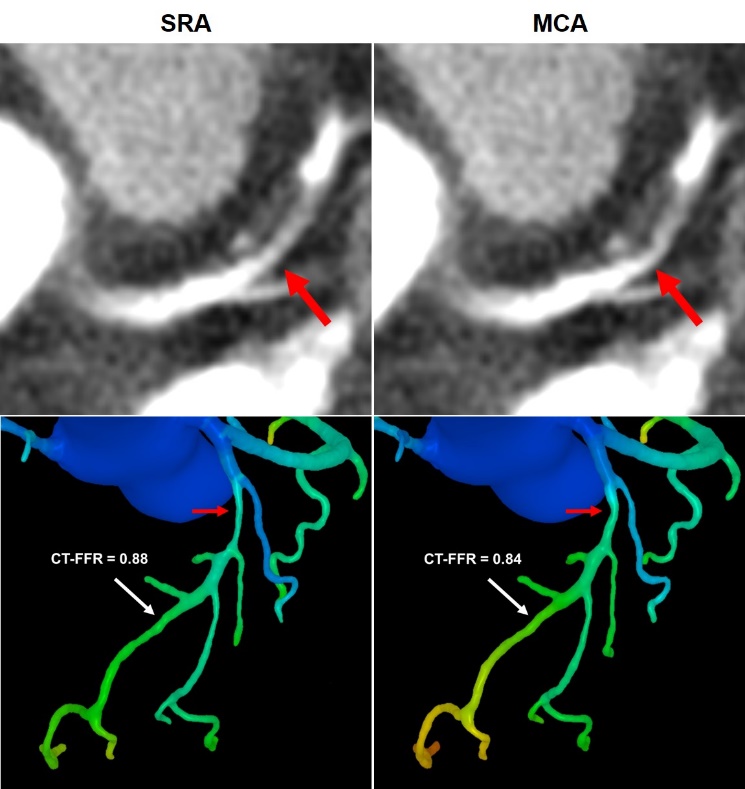


Fig. S1 Vascular distortions (red arrowhead) were found in one case after the use of MCA. CT-FFR (white arrowhead) were similar between images reconstructed with and without MCA.
